# Cefepime-induced encephalopathy: socio-clinical patterns and electroencephalographic findings

**DOI:** 10.1055/s-0045-1812469

**Published:** 2025-10-27

**Authors:** Vitor Roberto Pugliesi Marques, Lúcia Helena Neves Marques, Gerardo Maria de Araujo Filho, Nabila Darido Abdalla, Andressa Regina Galego, Vitor Brumato Fachini, Felipe Henrique Muniz, Breno Gonçalves Medeiros

**Affiliations:** 1 Faculdade de Medicina de São José do Rio Preto, São José do Rio Preto SP, Brazil.; 2 Hospital de Base de São José do Rio Preto, Centro de Cirurgia de Epilepsia, São José do Rio Preto SP, Brazil.; 3 Santa Casa de São Carlos, São Carlos SP, Brazil.; 4 Universidade Federal de São Paulo, São Paulo SP, Brazil.; 5 Faculdade de Medicina de São José do Rio Preto, Departamento de Ciências Neurológicas, Psiquiatria e Psicologia Médica, São José do Rio Preto SP, Brazil.

**Keywords:** Brain Diseases, Cefepime, Electroencephalography, Health Profile, Infections

## Abstract

**Background:**

Cefepime is an antibiotic widely used for severe infections in hospital . However, its use can lead to encephalopathy, which is detected by electroencephalogram (EEG).

**Objective:**

To establish the socioclinical pattern of cefepime encephalopathy and its correlation with EEG.

**Methods:**

Forty-one medical records of patients diagnosed with cefepime-induced encephalopathy were analyzed according to the criteria established by Naranjo et al., ^1^ with socioclinical parameters being evaluated.

**Results:**

All EEG tracings in the presence of cefepime-induced encephalopathy had generalized periodic discharges (GPD), and 70.7% of the exams met the criteria for a nonconvulsive status epilepticus. With the withdrawal of cefepime, 85.3% of patients had clinical improvement.

**Conclusion:**

Encephalopathy caused by cefepime is a clinical manifestation that should be considered among patients using this antibiotic, with a wide spectrum of manifestations. The use of EEG imaging is critical for diagnosis.

## INTRODUCTION

Encephalopathy is a clinical syndrome characterized by an altered mental state, affecting cognition or level of arousal, and can result from a variety of causes. 2 Conditions such as exposure to toxins, drugs, metabolic disturbances, and infectious diseases can contribute to encephalopathy and, among the drugs that can cause encephalopathy, cefepime must be cited. 2 3 Myoclonic seizures, hallucinations, asterixis, and generalized epileptic seizures have been associated to the use of cephalosporins, which are β-lactam antibiotics, and these manifestations may be a result of the encephalopathy caused by these drugs. 4 5 6

The potential for neurological complications and seizures resulting from the use of cefepime should not be neglected. In June 2012, the United States' Food and Drug Administration (FDA) issued a warning about the need to adjust its dose in patients with renal failure due to the risk of seizures and nonconvulsive status epilepticus. 7

Neurological manifestations such as mental confusion, agitation, disorientation, hallucinations, depressed level of consciousness, aphasia, asterixis, myoclonus, and seizures are associated with cefepime-induced encephalopathy. 8 9 10 11 12 13 Symptoms may appear between the 2 ^nd^ and 10 ^th^ day of drug use. 8 9 12 13 14 15 16 17

The present cross-sectional study aims to establish the clinical and electroencephalographic profile of encephalopathy caused by cefepime in a tertiary hospital, correlating electroencephalographic patterns with clinical symptoms.

## METHODS

The present retrospective observational study analyzed medical records of patients who used cefepime in a tertiary hospital between June 2015 and December 2017 and was approved by the local Research Ethics Committee.

To be eligible for the study, patients needed an electroencephalogram (EEG) showing alterations suggestive of encephalopathy, with cefepime as the only cause. Because of this, all patients presenting any confounding factors were excluded. All patients with metabolic conditions (e.g. electrolyte abnormalities, hyper- or hypoglycemia, and uremia) or using medications that could cause similar alterations in EEG (e.g., opioids and benzodiazepines) were excluded from the research.

The dependent variables analyzed included clinical manifestations (agitation, decreased consciousness level, epileptic manifestations) and EEG findings (slowing EEG and generalized periodic discharges [GPD]). The independent variables included socioeconomic characteristics, cefepime dose, renal function, and time to symptom onset.

The diagnostics of encephalopathy were based on EEG performed according to the international 10 to 20 system, and they should show signs of established encephalopathy, such as diffuse background abnormalities and GPDs. Data were presented using descriptive statistics (absolute and relative frequencies, measures of central tendency) and analyzed using inferential statistics, such as the Chi-squared test, to assess significant associations between variables, considering a 5% significance level.

### Data collection and inclusion and exclusion criteria

We analyzed patients who used cefepime between June 2015 and December 2017, through their medical record data. Patients who underwent EEG while using the drug were included, excluding those with other possible causes of encephalopathy, such as metabolic conditions and other drugs. Patients with significant alterations in serum levels of sodium, potassium, glycemia, liver enzymes, and uremia, as well as those with elevated infectious parameters, were excluded, along with those using opioids, benzodiazepines, and barbiturates.

#### *Naranjo et al.'s algorithm*

In 1981, Naranjo et al. 1 established criteria for verifying the cause-effect relationship of a given substance. The authors classified the causal relationship according to the total score as defined (≤ 9), probable (5–8), possible (1–4), and doubtful (≥ 0). Higher scores indicated a greater probability for reaction.

When this algorithm is used, the sample is selected according to the parameters defined by the authors to precisely establish the cause-effect relationship.

### Analysis of socioclinical data

Variables such as age, gender, race, profession, time to symptom onset, average dose used, and clinical indication for the use of cefepime were analyzed. Professions were defined according to the three sectors of the economy: rural, industry, and service, in addition to the retiree category. Patients' renal function was obtained through the Cockcroft-Gault 18 equations 19 and the Modification of Diet in Renal Disease (MDRD). 19 There was agreement between both when characterizing the study population into four categories: category 1 with a glomerular filtration rate ≥ 60 ml/min/1.73 m ^2^ ; category 2 with 30 to 59 ml/min/1.73 m ^2^ ; category 3 with 15 to 29 ml/min/1.73 m ^2^ ; and category 4 with ≤ 15 ml/min/1.73 m ^2^ . The decision to assess renal function using two methodologies was made to ensure that, regardless of the method employed, patients would be classified within the same range.

The onset of encephalopathy symptoms was determined based on the date of the EEG as, in the study's hospital, it is performed on the same day the clinician suspects a pathological condition. The clinical manifestations were classified into three: patients with 1. symptoms of agitation; 2. decreased levels of consciousness; and 3. epileptic manifestations.

### Electroencephalographic data analysis

The EEG reports were categorized as normal, slowing, or presence of GPD. The EEG exams lasted 30 minutes or more and were conducted according to the standardization of the international system. 7 8 9 10 11 12 13 14 15 Control EEGs were performed 48 hours after the discontinuation of cefepime, maintaining the same evaluation criteria.

The EEGs were classified as normal when they showed organized and symmetrical baseline electrical activity with an alpha rhythm in the posterior regions of the scalp, at a frequency of 8 to 12 Hz. Slowing EEG had a disorganized background activity with the presence of theta and/or delta band (theta: 4–7 Hz; delta: 0.1–3 Hz) predominant in its recording. For those with GPD, there was the presence of bilateral synchronous and symmetric periodic discharges. All EEG recordings were reviewed by specialized neurophysiologists and were analyzed according to the guidelines established by the American Clinical Neurophysiology Society (ACNS), published in 2021. 7 8 9 10 11 12 13 14 15

In the control EEG, in addition to the previously established criteria, the category of baseline rhythm alteration was included, characterized by a poor differentiation of anteroposterior rhythms without meeting the criteria for a slowing EEG. In all tracings, the presence of electrographic status epilepticus was evaluated according to the medical literature for nonconvulsive status epilepticus. 20

The frequencies obtained from the variables studied were analyzed using the Chi-squared test at 5% significance. It is noteworthy that none of the patients included in this study used anti-crisis medications, which could be responsible for their clinical improvement.

## RESULTS

**Figure 1:**
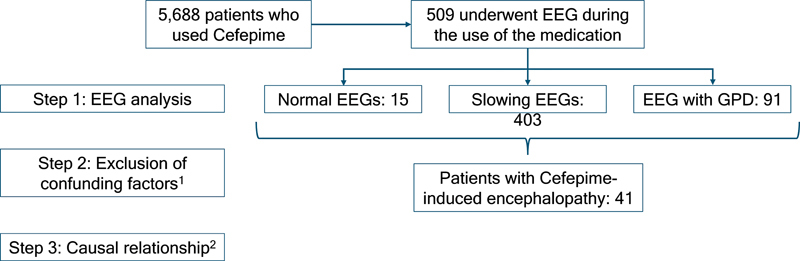
Flowchart of the patient inclusion criteria. ^1^ Confounding factors: imbalances in sodium, potassium, glycemia, liver function, uremia, and high parameters. ^2^ Classified into the “probable” and “possible” categories, according to Naranjo et al. 1981. 1

Among 5,688 patients who used cefepime, 509 underwent EEG during the use of the medication. The EEG patterns observed were analyzed in detail, with the following results observed: 15 had normal EEGs, 403 slowing EEG, and 91 had GPD. The clinical manifestations of all patients with abnormal EEGs were evaluated to determine whether they corresponded to the clinical features described in cefepime-induced encephalopathy. At the end of the analysis, a total of 41 cases were identified as having cefepime-induced encephalopathy. The flowchart of the patient inclusion criteria is in Figure 1 .

The causal relationship between cefepime and encephalopathy was considered strong, although no patient was classified as “defined” due to the cross-sectional nature of the study, without the use of placebos, antagonists, or serum dosage. 1 The Naranjo methodology was used to assess the likelihood of adverse reactions by applying the recommended criteria to assign possible, probable, or definite categories, based on the correlation between drug use and patients' clinical presentation.

The studied sample of 41 patients represented 0.72% of those who used cefepime during the study period. Additionally, an analysis of the socioeconomic conditions of the patients was performed ( Table 1 ).

**Table 1:** Socioeconomic analysis of the patients

Age (years)		73.83 ± 18.90
**Sex (%)**	Male	10 (24.4%)
	Female	31 (75.6%)
**Race (%)**	White	36 (87.8%)
	Black	3 (7.3%)
	Mixed	2 (4.9%)
**Profession (%)**	Rural	6 (14.6%)
	Industry	4 (9.8%)
	Services	20 (48.8%)
	Retired	11 (26.8%)

Source: Prepared by the authors.

As shown in the table, the mean age confidence interval was 73.83 ± 18.90-years, with a relative frequency predominance of females (75.6%), white individuals (87.8%), and professionals from the service sector (48.8%).

Cefepime was primarily indicated for pneumonia (65.9%), followed by urinary tract infection (12.2%), febrile neutropenia (9.8%), soft tissue infection (4.8%), intra-abdominal infection (2.4%), and other infections, such as in the bloodstream (4.8%). The average time to symptom onset ranged from 2.48 to 8.2 days. Clinically, a decrease in the level of consciousness was observed in 80.5% of the patients, epileptic manifestations in 12.2%, and agitation in 7.3%. Table 2 correlates the indication of cefepime with the clinical symptoms observed.

**Table 2:** Indication for the use of cefepime and clinical manifestations

Indication of Cefepime	Clinical condition of patient			
	Agitation	LLC	Epileptic seizures	Total
Pneumonia	1 (2.4%)	22 (53.7%)	4 (9.8%)	27 (65.9%)
ICU	0 (0%)	4 (9.8%)	1 (2.4%)	5 (12.2%)
Intra-abdominal	0 (0%)	1 (2.4%)	0 (0%)	1 (2.4%)
Febrile neutropenia	1 (2.4%)	3 (7.3%)	0 (0%)	4 (9.8%)
Soft tissues	0 (0%)	2 (4.8%)	0 (0%)	2 (4.8%)
Others	1 (2.4%)	1 (2.4%)	0 (0%)	2 (4.8%)
Total	3 (7.3%)	33 (80.5%)	5 (12.2%)	41 (100%)

Abbreviations: ICU, intensive care unit; LLC, lowered level of consciousness.

Source: Prepared by the authors.

An evaluation of the possible relationship between the indication of cefepime and the clinical manifestation of encephalopathy was performed using the Chi-squared test. The *p* -value obtained was 0.472, indicating that there is no significant association between the site of infection and encephalopathy. Thus, it was concluded that this condition should be considered in all patients using the drug, regardless of the site of infection.

The average creatinine level was 1.66 ± 0.96. The distribution of renal dysfunction was as follows: 13 patients (31.7%) in category 1, 9 (22.0%) in category 2, 14 (34.1%) in category 3, and 5 patients (12.2%) in category 4. The average dose of cefepime was 4.14 ± 1.81, and 15 patients (36.6%) received a dose that was not adjusted according to renal function.

All EEG patterns in patients with cefepime-induced encephalopathy showed the presence of GPD, with 29 (70.7%) of them meeting criteria for nonconvulsive status epilepticus. The imaging test showed the presence of GPDs at the frequency between 1 and 4 Hz, with background disorganization. The tracings that met criteria for nonconvulsive status epilepticus were those presenting discharges at a frequency > 2.5 Hz, or those with discharges < 2.5 Hz accompanied by at least one of the three following findings: ictal phenomena at the time of the recording; or clinical and electrographic improvement following administration of an anti-seizure medication (1 mg of intravenous midazolam); or modification of the tracing in terms of time and space throughout the recording.

There were no normal results, nor any classified only as a slowing EEG in the first analysis. Control tests were performed in 26 patients (63.4%), revealing 3 (7.3%) with baseline rhythm alterations, 18 (43.9%) with a slowing EEG, 2 (4.9%) with persistent GPD, and 2 patients (4.9%) with persistent nonconvulsive status epilepticus. The clinical outcome of the patients (improved, discharge, and death) was verified after cefepime withdrawal, as shown in Table 3 .

**Table 3:** Analysis of the association between evolution after cefepime withdrawal and clinical outcome

Evolution after cefepime	Clinical outcome		
	Improvement and discharge	Death	Total
Improvement	20 (48.8%)	15 (36.5%)	35 (85.3%)
No change	0 (0.0%)	2 (4.9%)	2 (4.9%)
Worsening	0 (0.0%)	4 (9.8%)	4 (9.8%)
Total	20 (48.8%)	21 (51.2%)	41

Source: Prepared by the authors.

The evolution after the withdrawal of cefepime and the clinical outcome among the different groups was analyzed according to the Chi-squared test statistic of 6.694 and a *p* -value of 0.035, and there was statistical evidence of an association for the hypothesis of evolution. Among the 85.3% of patients who showed clinical improvement, 48.8% had favorable and 36.5% had unfavorable outcomes, progressing to death. Meanwhile, among the 4.9% of patients with no change and the 9.8% who had worsening of the clinical condition, none had the favorable outcome of improved discharge, and all these patients progressed to death. The EEG improvement of most patients (51.2%) documents the improvement in cortical involvement with the withdrawal of cefepime.

### Study's limitations

This study presents several limitations that should be considered when interpreting the results. Conducting the study in a single tertiary hospital limits the generalizability of the findings to other institutions or populations. Additionally, the exclusion of patients with other potential causes of encephalopathy, such as metabolic imbalances and severe infectious conditions, may have led to underrepresentation of relevant cases.

The use of retrospective medical records introduces the possibility of incomplete or biased data, compromising the accuracy of the collected information. The small sample size, with only 41 patients, limits the statistical power of the conclusions, potentially underestimating or overestimating the prevalence of cefepime-associated encephalopathy.

The absence of a comparable control group complicates the clear delineation of cefepime's specific effects relative to other clinical factors. Furthermore, coexisting medical conditions and the concurrent use of other medications may have acted as confounding factors in the observed results.

Despite these challenges, the present study's findings underscore the importance of considering encephalopathy as a potential adverse effect of cefepime use, particularly in elderly patients or those with comorbidities. Future studies with larger samples and prospective approaches are needed to confirm and expand these results.

## DISCUSSION

Of the 5,688 patients in the study who used cefepime, 509 underwent EEG, and 41 (0.72%) presented with encephalopathy. The high prevalence in the elderly confirms data from the literature that this population is more affected. 21

The number of patients with cefepime-induced encephalopathy might be higher than what was identified. This is due to two factors: first, patients with other associated clinical conditions, such as imbalances in sodium, potassium, glycemia, liver function, uremia, and high infectious parameters were excluded from the study, as these conditions often coexist in the context of cefepime use. Second, encephalopathy as a side effect of this drug is not well known, resulting in lower clinical suspicion and, therefore, fewer EEG requests, which is the essential diagnostic tool.

No correlation was found between the site of infection and encephalopathy, indicating that this condition should be considered for all patients using the drug. Patients with altered renal function were the most affected, corresponding to 68.3% of the sample, confirming data from the literature. 21

The evaluation of the cefepime dose in relation to renal function revealed that in 36.6% of the cases the dose was not adjusted according to renal function, indicating a high rate of inadequacy in correction. This data is important for physicians, as medical literature emphasizes that adjusting medication doses based on renal function is crucial for nephroprotection and patient safety. 22

All samples presented GPD, and 70.7% of the exams indicated nonconvulsive status epilepticus. This pattern of discharges is described in medical literature about cefepime-induced encephalopathy, especially in elderly patients and those with renal failure, and was also present in this study.

There are reports in the medical literature of unfavorable outcomes in patients using cefepime. 21 In this study, the favorable outcome of improvement and discharge was observed only in the group that showed clinical improvement after medication withdrawal. This highlights the importance of rigorous vigilance to identify possible manifestations of encephalopathy, allowing for appropriate intervention, which in this case is the withdrawal of the drug.

In conclusion, cefepime-induced encephalopathy should be considered in this cohort, as it can manifest in various forms, ranging from agitation to decreased level of consciousness and epileptic manifestations. The use of EEG is crucial for the diagnosis, helping to differentiate it from other conditions. The high prevalence among elderly patients and those with renal dysfunction confirms previous findings in the medical literature.

The identification of cefepime-induced encephalopathy and its treatment, through drug withdrawal or change, showed that 85.3% of patients improved, with 48.8% presenting favorable outcomes, indicating that this condition should not be neglected.

It can be stated with 5% significance that the indication of cefepime and patients' clinical condition have no correlation. On the other hand, the withdrawal of the medication in comparison to the clinical outcome statistically ensures that there is significant patient improvement.

The high prevalence among elderly patients and those with renal dysfunction confirms previous data. The identification and treatment of cefepime-induced encephalopathy, with the withdrawal of the drug, led to significant improvement in patients, indicating the need for attention from healthcare professionals.
